# The association between body fatness and mortality among breast cancer survivors: results from a prospective cohort study

**DOI:** 10.1007/s10654-023-00979-5

**Published:** 2023-03-29

**Authors:** Catalina Bonet, Marta Crous-Bou, Konstantinos K. Tsilidis, Marc J. Gunter, Rudolf Kaaks, Matthias B. Schulze, Renée T. Fortner, Christian S. Antoniussen, Christina C. Dahm, Lene Mellemkjær, Anne Tjønneland, Pilar Amiano, Eva Ardanaz, Sandra M. Colorado-Yohar, Miguel Rodriguez-Barranco, Sandar Tin Tin, Claudia Agnoli, Giovanna Masala, Salvatore Panico, Carlotta Sacerdote, Anne M. May, Kristin Benjaminsen Borch, Charlotta Rylander, Guri Skeie, Sofia Christakoudi, Dagfinn Aune, Elisabete Weiderpass, Laure Dossus, Elio Riboli, Antonio Agudo

**Affiliations:** 1 Unit of Nutrition and Cancer, Epidemiology Research Program, Catalan Institute of Oncology—ICO, L’Hospitalet de Llobregat, Spain; 2 Nutrition and Cancer Group, Bellvitge Biomedical Research Institute—IDIBELL, Av. Granvia de L’Hospitalet 199-203, 08908 L’Hospitalet de Llobregat, Spain; 3 Department of Epidemiology, Harvard T.H. Chan School of Public Health, Boston, MA 02115 USA; 4 Department of Epidemiology and Biostatistics, School of Public Health, Imperial College London, London, UK; 5 Department of Hygiene and Epidemiology, University of Ioannina School of Medicine, Ioannina, Greece; 6 Nutrition and Metabolism Branch, International Agency for Research on Cancer (IARC/WHO), Lyon, France; 7 Division of Cancer Epidemiology, German Cancer Research Center (DKFZ), Heidelberg, Germany; 8 Department of Researh, Cancer Registry of Norway, Oslo, Norway; 9 Department of Molecular Epidemiology, German Institute of Human Nutrition Potsdam-Rehbruecke, Nuthetal, Germany; 10 Institute of Nutritional Science, University of Potsdam, Nuthetal, Germany; 11 Department of Public Health, Aarhus University, 8000 Aarhus C, Denmark; 12 Danish Cancer Society Research Center, Diet, Genes and Environment, Copenhagen, Denmark; 13 Sub Directorate for Public Health and Addictions of Gipuzkoa, Ministry of Health of the Basque Government, 2013 San Sebastian, Spain; 14 Epidemiology of Chronic and Communicable Diseases Group, Biodonostia Health Research Institute, 20014 San Sebastian, Spain; 15 CIBER of Epidemiology and Public Health (CIBERESP), Madrid, Spain; 16 Navarra Public Health Institute, Pamplona, Spain; 17 IdiSNA, Navarra Institute for Health Research, Pamplona, Spain; 18 Department of Epidemiology, Murcia Regional Health Council, IMIB-Arrixaca, Murcia, Spain; 19 Research Group on Demography and Health, National Faculty of Public Health, University of Antioquia, Medellín, Colombia; 20 Escuela Andaluza de Salud Pública (EASP), 18011 Granada, Spain; 21 Instituto de Investigación Biosanitaria Ibs.GRANADA, 18012 Granada, Spain; 22 Cancer Epidemiology Unit, Nuffield Department of Population Health, University of Oxford, Oxford, UK; 23 Epidemiology and Prevention Unit Department of Research, Fondazione IRCCS Istituto Nazionale Dei Tumori Via Venezian, 1-20133 Milan, Italy; 24 Institute for Cancer Research, Prevention and Clinical Network (ISPRO), Florence, Italy; 25 Dipartimento Di Medicina Clinica E Chirurgia, Federico II University, Naples, Italy; 26 Unit of Cancer Epidemiology, Città della Salute e della Scienza University-Hospital, Via Santena 7, 10126 Turin, Italy; 27 Julius Center for Health Sciences and Primary Care, University Medical Center Utrecht, Utrecht University, Utrecht, The Netherlands; 28 Department of Community Medicine, Faculty of Health Sciences, UiT The Arctic University of Norway, Tromsö, Norway; 29 Department of Inflammation Biology, School of Immunology and Microbial Sciences, King’s College London, London, UK; 30 Department of Nutrition, Oslo New University College, Oslo, Norway; 31 Department of Endocrinology, Morbid Obesity and Preventive Medicine, Oslo University Hospital, Oslo, Norway; 32 Director Office, International Agency for Research on Cancer, World Health Organization, Lyon, France

**Keywords:** Body fatness, Weight change, Breast cancer survivors, Mortality, Breast cancer-specific mortality, Prospective study

## Abstract

Evidence linking body fatness to breast cancer (BC) prognosis is limited. While it seems that excess adiposity is associated with poorer BC survival, there is uncertainty over whether weight changes reduce mortality. This study aimed to assess the association between body fatness and weight changes pre- and postdiagnosis and overall mortality and BC-specific mortality among BC survivors. Our study included 13,624 BC survivors from the European Prospective Investigation into Cancer and Nutrition (EPIC) study, with a mean follow-up of 8.6 years after diagnosis. Anthropometric data were obtained at recruitment for all cases and at a second assessment during follow-up for a subsample. We measured general obesity using the body mass index (BMI), whereas waist circumference and A Body Shape Index were used as measures of abdominal obesity. The annual weight change was calculated for cases with two weight assessments. The association with overall mortality and BC-specific mortality were based on a multivariable Cox and Fine and Gray models, respectively. We performed Mendelian randomization (MR) analysis to investigate the potential causal association. Five-unit higher BMI prediagnosis was associated with a 10% (95% confidence interval: 5–15%) increase in overall mortality and 7% (0–15%) increase in dying from BC. Women with abdominal obesity demonstrated a 23% (11–37%) increase in overall mortality, independent of the association of BMI. Results related to weight change postdiagnosis suggested a U-shaped relationship with BC-specific mortality, with higher risk associated with losing weight or gaining > 2% of the weight annually. MR analyses were consistent with the identified associations. Our results support the detrimental association of excess body fatness on the survival of women with BC. Substantial weight changes postdiagnosis may be associated with poorer survival.

**Supplementary Information:**

The online version contains supplementary material available at 10.1007/s10654-023-00979-5.

## Introduction

In 2020, female breast cancer (BC) was the most common cancer worldwide, with 2.3 million new cases and 685,000 BC-related deaths [1]. Evidences from observational studies suggest that obesity is associated with a 14–26% increased risk of recurrence and BC-specific mortality [2]. This finding has been firmly established for estrogen receptor-positive (ER +) BCs [3]. An expert panel reported that there is strong evidence to suggest elevated body fatness is a predictor of poor outcome in BC survivors; however, the impact of weight changes or specific weight loss in obese or overweight survivors of BC on reduced mortality is uncertain [4]. Excess body fat poses a challenge for the clinical management of BC; therefore, the oncologists have identified the assessment of its role in survival and prognosis as a research priority [5].

The majority of results supporting the evidence for the association between higher body mass index (BMI) and survival following BC are obtained from case series or cohorts of patients selected from a clinical setting, including trial participants [4, 6]. These studies have the strength of providing relevant clinical information; however, they are based upon highly selected samples. Few results come from population-based prospective studies. Despite lacking detailed information on clinical features, they provide a broader spectrum of the whole population of BC survivors.

Our primary objective was to assess the association between body fatness and weight changes (pre- and postdiagnosis) and the overall mortality and BC-specific mortality among BC survivors, using data from the European Prospective Investigation into Cancer and Nutrition (EPIC) cohort. Subsequently, we aimed to conduct a Mendelian randomization analysis to investigate the potential causal role of BMI in survival.

## Methods

### Study design and participants

The EPIC is a multi-center cohort study comprising 521,330 participants across 10 European countries. Recruitment procedures and data collection of the EPIC study have been described elsewhere [7]. We collected information on the diet, lifestyle factors, anthropometric measurements, medical history, and blood samples at baseline. Moreover, we provided additional questionnaires to measure lifestyle changes few years following the recruitment for a subset of participants [8]. All volunteers provided informed consent at recruitment. The study was approved by the ethical committees from the International Agency for Research on Cancer and the national centers.

Our study included available data of women from nine of the participant countries (France, Italy, Spain, United Kingdom, The Netherlands, Germany, Sweden, Denmark, and Norway), diagnosed with primary BC during follow-up.

### Breast cancer diagnosis and vital status ascertainment

In the majority of participating countries, incident BC cases and vital status were assessed via record linkages to the regional or national cancer registries and national mortality registries. In France and Germany, cancer cases were identified based on cancer and pathology registries, health insurance records, and an active follow-up by contacting the participants or their next-of-kin. The follow-up for cancer endpoints, vital status, and the originating causes of death are available until 2015.

BCs were defined as tumors coded C50.0–50.9 in the International Classification of Diseases for Oncology [9]. Only primary malignant neoplasms were considered. A total of 13,686 incident BC cases were identified, following the exclusion of nine, 20, and 33 cases with an unknown vital status and the date of death or censoring, without follow-up data, and non-epithelial or mixed breast tumors, respectively. Eventually, 13,624 patients with BC were analyzed. The information on histology and tumor receptor status, gathered from pathology reports, was available for 30–70% of the cases, depending on the centers.

### Data collection and variables

Baseline anthropometry was measured with standardized procedures in the majority of centers, except for those of Oxford (UK), France, and Norway, which collected self-reported data [10]. The second anthropometric assessment was performed at an average of 5 years following the recruitment of 10,277 cases; weight was self-reported in all centers, except Norfolk (UK), which measured the weight and height.

The BMI (kg/m^2^) was the primary indicator of general obesity. Waist circumference (WC) and A Body Shape Index (ABSI) [11] were the measures of abdominal obesity. For BC cases with two weight assessments, we calculated the annual weight change (kg/year) as the difference between the two weight measures divided by the number of years between the assessments. It was expressed as percentages (%), with respect to the baseline weight.

Information on educational level, physical activity, smoking habits, alcohol intake, and menopause status was collected using lifestyle questionnaires at baseline.

### Statistical analysis

We estimated the hazard ratios (HRs) and 95% confidence intervals (CI) to determine the association between body fatness or weight change and the overall mortality using Cox Proportional Hazard models. For BC-specific mortality, we used the Fine and Gray subdistribution hazard models [12], which considered other causes of death as a competing event. The entry time was defined as the date of diagnosis for analyzing the prediagnosis anthropometry measures or the date at the second weight assessment for analyzing the postdiagnosis measures; the exit time was defined as the date at death, emigration, or the end of follow-up. The survival models were stratified by country and menopausal status at diagnosis, and adjusted for potential confounders as follows: age at diagnosis, the level of education, physical activity, alcohol consumption, smoking habit, the use of hormone therapy for menopause, tumor stage, tumor grade, and tumor receptor status. We evaluated the model assumptions with graphs and tests based on the scaled Schoenfeld residuals. Detailed information on the applied models and the definition and handling of covariates are outlined in the supplementary material.

### Analyzing prediagnosis body fatness

We constructed the survival models for each of the three baseline measures of obesity independently as continuous and categorical variables, and combined the two uncorrelated indices of general and abdominal obesity, namely BMI and ABSI [11]. The BMI and WC at recruitment were categorized based on standard cut-offs (BMI: Underweight < 18.5 kg/m^2^, Normal weight 18.5–24.9 kg/m^2^, Overweight 25–29.9 kg/m^2^, Obese ≥ 30 kg/m^2^; WC: ≤ 88 cm and > 88 cm for a very high waist circumference) [13], whereas the ABSI was standardized by a *z*-score when used as a continuous variable. We used the 75th percentile of the distribution (76.04) to dichotomize participants into high and low ABSI [14]. For continuous variables, we used restricted cubic splines models [15] to determine the validity of the assumed log-linear dose–response association with the mortality outcomes. Moreover, we assessed the non-linearity using the Likelihood Ratio (LR) test. The direct-adjusted survival and cumulative incidence estimates by BMI categories were derived from the Cox and Fine and Gray models [16]. We performed subgroup analyses by the menopausal status, stage, and the receptor status of the tumor; the heterogeneity across groups was determined using the LR test.

We performed several sensitivity analyses to confirm the results obtained from the primary analyses of body fatness and mortality. Since we assumed that the BMI at baseline remained constant until diagnosis, we first conditioned the multivariable Cox model for the overall mortality for different time-periods before diagnosis, and second, we re-ran the survival models adjusting by time from baseline to diagnosis. Furthermore, we adjusted the multivariable Cox model for the time period of diagnosis to consider differences in the availability of treatments over the follow-up.

### Analyzing postdiagnosis body fatness

We included a subset of 1878 BC cases that underwent a second weight assessment within 6 months and 4 years postdiagnosis. The postdiagnosis BMI was calculated using the height recorded at recruitment. We used the similar multivariable and stratified the Cox and Fine and Gray models, with additional adjustments by the prediagnosis BMI and the time from diagnosis to the second weight assessment.

### Analyzing weight change

The percentage (%) of annual weight change was considered for the analyses and grouped into three categories as follows: weight loss (< − 0.6%), stable weight (− 0.6 to 0.6%), and weight gain (> 0.6%) [17]. We conducted separate analyses by determining if the second weight assessment was performed pre- or postdiagnosis. Furthermore, separate analyses were performed for the baseline BMI < 25 kg/m^2^ and ≥ 25 kg/m^2^ for prediagnosis weight change. The models assessing the postdiagnosis weight change were further adjusted by the prediagnosis BMI.

### Mendelian randomization analysis

We used genome-wide association study data available for 8494 participants, which included 3830 BC cases. We constructed a weighted genetic risk score (wGRS), comprising 94 single nucleotide polymorphisms related with the BMI for women reported in the GIANT consortium [18]. We estimated the association between the BMI and wGRS with a linear regression model in non-cases and the association between the mortality and wGRS with a Cox proportional hazards model in cases. Subsequently, we used an instrumental variable ratio estimate to assess the causal relationship between the BMI and overall and BC mortality [19]. Additional information on the datasets, genotyping procedures, imputation method, the two-sample MR analysis, and results is provided in the supplementary material.

We used the SAS version 9.4 for the survival analyses and R software for the MR analyses [20].

### Patient and public involvement

The scientific research conducted in this article corresponds to concerns of the woman involved in the EPIC cohort. The results of the present study will be disseminated through institutional websites and the media.

## Results

### Baseline characteristics

The 13,624 BC survivors had a mean follow-up of 8.6 years (SD 4.9 years). During the follow-up, 2425 women died, of which 1354 of breast cancer. The country-based distribution of the survivors and deaths, and the list of causes of death are provided in the supplemental material (Supplementary Tables 1, 2). A total of 13% of BCs were metastatic at diagnosis, and more than half of them were ER + (Table 1. Supplementary Table 3). The average age at diagnosis was 61.1 years (78% postmenopausal); 19% were current smokers, 27% consumed one or more drinks of alcohol per day (> 12gr of ethanol/day). In addition, more than half were inactive or moderately inactive (57%). The average of the primary anthropometric measurements were 24.9 kg/m^2^ for BMI, 80.3 cm for WC, and 73.0 for the ABSI; 29.7% and 11.5% participants were overweight and obese (Supplementary Table 4).

**Table 1:** Baseline and tumor characteristics of the breast cancer survivors

	All women *N* = 13,624
*Demographic and lifestyle factors*	*N(%)*
*Highest school level*	
None/primary school completed	3400 (26.2)
Technical/professional school	3121 (24.1)
Secondary school	3255 (25.1)
Longer education (including university degree)	3196 (24.6)
*Smoking status and intensity of smoking*	
Never	6086 (44.7)
Current, 1–15 cig/day	1636 (12.0)
Current, 16–25 cig/day	768 (5.6)
Current, 26 + cig/day	136 (1.0)
Former, quit < = 10 years	1086 (8.0)
Former, quit 11–20 years	1013 (7.4)
Former, quit 20 + years	1125 (8.3)
Miscellaneous	1515 (11.1)
*Alcohol consumption gr/day*	
Non drinker	1830 (13. 6)
> 0–3	3889 (28.8)
> 3–12	4119 (30.5)
> 12–24	2086 (15.5)
> 24	1570 (11.6)
*Physical activity*	
Inactive	2784 (20.8)
Moderately inactive	4824 (36.0)
Moderately active	3674 (27.4)
Active	2117 (15.8)
*Menopausal status at diagnosis*	
Premenopausal	2945 (21.6)
Postmenopausal	10,679 (78.4)
*Ever taken menopausal hormone therapy*	
No	7682 (56.4)
Yes	5449 (40.0)
*Clinical and histological features of the tumor*	*N(%)*
*Grade*	
Well differentiated	1323 ( 9.7)
Moderately differentiated	2991 (21.9)
Poorly differentiated/Undifferentiated	2549 (18.7)
Not determined	6761 (49.6)
*Stage*	
Stage 0/I	1984 (14.6)
Stage II	1624 (11.9)
Stage III	312 ( 2.3)
Non-metastatic unknown stage^1^	4101 (30.1)
Stage IV (metastatic)	1814 (13.3)
Unknown	3789 (27.8)
*Estrogen receptor status*	
Negative	1718 (12.6)
Positive	7723 (56.7)
Unknown	4183 (30.7)
*Progesterone receptor status*	
Negative	2676 (19.6)
Positive	5183 (38.0)
Unknown	5765 (42.3)
*Human epidermal receptor 2 status*	
Negative	3679 (27.0)
Positive	870 ( 6.4)
Unknown	9075 (66.6)

The number of missing observations for Highest school level, Smoking status and intensity of smoking, Alcohol consumption, Physical activity and Ever taken menopausal hormone therapy were 652, 259, 130, 225 and 493, respectively

^1^Non-metastatic unknown stage category includes all tumors without distant metastases (M0) with insufficient information on the size of the tumor (T) and regional lymph node status (N), to be classified within the specific stage I, II or III

### Prediagnosis body fatness and mortality

High BMI, WC, and ABSI measured prior to the diagnosis were associated with an increasing risk of overall mortality when assessed independently (Table 2); we observed increases of 10% (95%CI: 5–15%), 11% (6–16%), and 9% (4–14%) for each 5-kg/m^2^ of BMI, 10 cm of WC, and per 1-SD increase in ABSI, respectively. Upon including the BMI and ABSI in the same model, they displayed association with mortality: women with obesity revealed an increase in the overall mortality by 25% (9–43%), compared with women with normal weight; those with abdominal obesity (high vs low ABSI) revealed an increase of 22% (10–35%). These findings appear to be limited to postmenopausal women. Despite similar estimates for premenopausal women, none of the associations were statistically significant. The BMI displayed a J-shaped association with the overall mortality, with a *p*-value of 0.077 when testing deviation from linearity (Supplementary Fig. 1).

**Table 2:** Association of anthropometric measures with overall and breast cancer-specific mortality

	Overall mortality		Breast cancer mortality	
	HR^a^	95% CI	HR^b^	95% CI
*Independent association of the anthropometric measurement*				
*Body Mass Index*^*1*^				
Underweight	1.10	(0.79–1.52)	1.25	(0.79–1.97)
Overweight	1.09	(0.99–1.19)	1.13	(1.00–1.28)
Obesity	1.26	(1.12–1.43)	1.23	(1.04–1.46)
Per 5 kg/m^2^ increase	1.10	(1.05–1.15)	1.07	(1.00–1.15)
*Waist Circumference*^*2*^				
> 88 cm	1.25	(1.12–1.40)	1.17	(1.01–1.35)
Per 10 cm increase	1.11	(1.06–1.16)	1.07	(1.01–1.14)
*ABSI*^*2*^				
High	1.23	(1.11–1.37)	1.13	(0.98–1.30)
Per 1 SD increase	1.09	(1.04–1.14)	1.04	(0.97–1.11)
*Model with Body Mass Index and ABSI*^*2*^				
*Body Mass Index*				
Underweight	1.29	(0.87–1.91)	1.35	(0.79–2.30)
Overweight	1.04	(0.94–1.16)	1.09	(0.95–1.25)
Obesity	1.25	(1.09–1.43)	1.23	(1.03–1.47)
*ABSI*				
High	1.22	(1.10–1.35)	1.11	(0.96–1.28)
*Premenopausal*^*3*^				
*Body Mass Index*				
Underweight	1.17	(0.54–2.53)	1.25	(0.50–3.11)
Overweight	1.06	(0.81–1.39)	0.98	(0.72–1.33)
Obesity	1.11	(0.77–1.61)	0.97	(0.63–1.49)
*ABSI*				
High	1.24	(0.94–1.63)	1.15	(0.83–1.59)
*Postmenopausal*^*4*^				
*Body Mass Index*				
Underweight	1.46	(0.92–2.32)	1.60	(0.83–3.08)
Overweight	1.04	(0.92–1.17)	1.11	(0.95–1.30)
Obesity	1.26	(1.09–1.46)	1.30	(1.06–1.59)
*ABSI*				
High	1.20	(1.07–1.35)	1.10	(0.94–1.28)

Abbreviations: ABSI, A Body Shape Index; HR, Hazard Ratio; CI, Confidence Interval

Reference levels for categorical variables: Body Mass Index - Normal weight 18.5-<25 kg/m^2^, Waist circumference <=88cm, ABSI - Low (<= 76.04, 75th Percentile)

^1^N = 13,624/2425 Deaths (1241 normal weight)/1354 BC deaths (665 normal weight)

^2^N = 9708/1850 Deaths (862 normal weight)/1111 BC deaths (511 normal weight)

^3^N = 1904/339 Deaths (207 normal weight)/261 BC deaths (163 normal weight)

^4^N = 7804/1511 Deaths (655 normal weight)/850 BC deaths (348 normal weight)

^a^Hazard Ratios from the multivariable Cox regression model

^b^Subdistribution Hazard Ratios from the multivariable Fine and Gray regression model

Regarding BC-specific mortality (Table 2), the associations for higher BMI and WC were significant when assessed independently, with effect estimates similar to those for the overall mortality. Contrarily, the ABSI revealed an attenuated (non-significant) association. Upon including the BMI and ABSI in the model, general obesity measured by the BMI was associated with a 23% (3–47%) increase in the BC-specific mortality. However, we didn’t observe an association for abdominal obesity (measured by high ABSI). The analysis by menopausal status generated similar results only for postmenopausal women. The assessment of linearity in the associations between BMI and BC-specific mortality (Supplementary Fig. 1) indicated a linear dose–response.

**Fig. 1:**
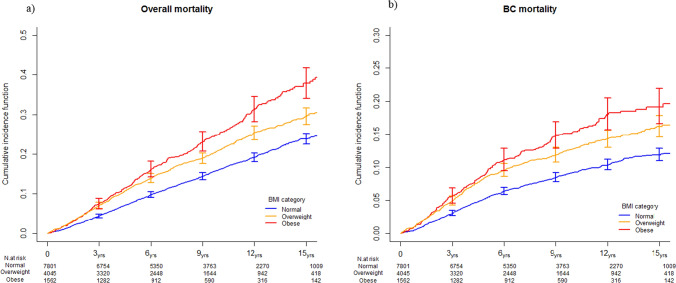
Non parametric estimates of the cumulative incidence curves for overall mortality (**a**) and breast cancer-specific mortality (**b**)

Figure 1 depicts the unadjusted cumulative incidence curves for the overall mortality and BC-specific mortality based on BMI categories. The graphs were consistent with the findings from the multivariable Cox and Fine and Gray models (Table 2) upon testing equality across the BMI categories (*p*-values < 0.0001). The 15-year overall and BC survival rates were 62% and 19%, 70% and 16%, and 76% and 12% for women who were obese, overweight, and with normal weight, respectively. We observed a similar pattern among postmenopausal women, without distinct differences across the BMI categories among premenopausal women (Supplementary Fig. 2).

The increases in overall mortality per five-unit increase in BMI were similar between women with metastatic and non-metastatic tumors, although the association was higher in the latter (Table 3). We observed higher mortality in women with ER + (15%, 7–23%), progesterone receptor-positive (PR +) (19%, 9–30%), and human epidermal growth factor receptor 2-negative (HER2-) (20%, 8–33%) tumors; however, only the estimates for PR + and PR- tumors displayed significant heterogeneity. The HRs for BC-specific mortality and BMI among ER + and HER2- tumors were 1.14 (1.03–1.25) and 1.16 (1.01–1.33) respectively; nonetheless, the heterogeneity tests by the ER and HER2 status did not achieve statistical significance.

**Table 3:** Association of 5-unit increase in Body Mass Index with overall and breast cancer-specific mortality, by characteristics of the tumor

		Overall mortality			Breast cancer mortality		
	N	Deaths	HR^a^	95% CI	Deaths	HR^b^	95% CI
*Stage*							
Metastatic	1814	604	1.06	(0.96–1.18)	377	1.11	(0.97–1.27)
Non-metastatic	8021	1005	1.18	(1.10–1.27)	516	1.11	(0.99–1.24)
Heterogeneity *p*-value				0.336			0.963
*Estrogen receptor status*							
ER +	7723	1124	1.15	(1.07–1.23)	533	1.14	(1.03–1.25)
ER-	1718	434	1.04	(0.92–1.18)	287	1.05	(0.89–1.24)
Heterogeneity *p*-value				0.051			0.135
*Hormone receptor status*							
PR +	5183	636	1.19	(1.09–1.30)	285	1.03	(0.89–1.18)
PR -	2676	529	1.04	(0.93–1.17)	311	1.09	(0.94–1.27)
Heterogeneity *p*-value				0.038			0.872
*HER2 status*							
HER2 +	870	167	1.05	(0.87–1.26)	116	0.92	(0.72–1.17)
HER2-	3679	488	1.20	(1.08–1.33)	276	1.16	(1.01–1.33)
Heterogeneity *p*-value				0.160			0.073

Abbreviations: HR, Hazard Ratio; CI, Confidence Interval

Heterogeneity test: Likelihood Ratio Test

^a^Hazard Ratios from the multivariate Cox regression model

^b^Subdistribution Hazard Ratios from the multivariable Fine and Gray regression model

The findings remained practically unchanged upon estimating the association between the BMI-measured body fatness and the overall mortality according to different time periods before diagnosis (Supplementary Table 5). We obtained similar results as those provided in Table 2, with additional adjustments by the time from baseline to diagnosis or by the period of diagnosis (Supplementary Tables 6, 7).

### Postdiagnosis body fatness and mortality

We observed higher risks for women with obesity than those women with a normal weight while analyzing the postdiagnosis BMI (32%, − 2–77%, and 18%, − 20–73%, for the overall mortality and BC-specific mortality, respectively) (Table 4). However, the estimates did not reach statistical significance probably because of the lack of power owing to fewer events. Upon considering the BMI as a continuous variable, we observed an increase of 13% (1–27%) in the overall mortality for each five-unit BMI increase.

**Table 4:** Association of post-diagnosis Body Mass Index with overall mortality and breast cancer-specific mortality

	Overall mortality		Breast cancer mortality	
	HR^a^	95% CI	HR^b^	95% CI
*Body mass index*				
Underweight	0.88	(0.35–2.20)	0.35	(0.06–2.04)
Overweight	1.03	(0.82–1.30)	0.79	(0.59–1.07)
Obesity	1.32	(0.98–1.77)	1.18	(0.80–1.73)
Per 5 kg/m^2^ increase	1.13	(1.01–1.27)	1.09	(0.93–1.28)
*% Annual weight change*				
Lowest tertile (< 0)	1.45	(1.13–1.87)	1.41	(0.99–2.00)
Highest tertile (≥ 0.96)	1.30	(1.00–1.67)	1.27	(0.91–1.79)

Abbreviations: HR, Hazard Ratio; CI, Confidence Interval

N = 1878/432 Deaths (214 normal weight) -/241 BC deaths (124 normal weight)

Weight assessment gathered between 6 months and 4 years post diagnosis

Reference level for categorical Body Mass Index: Normal weight 18.5-<25kg/m^2^

Reference level for % annual weight change: Middle tertile (0- < 0.96)

^a^Hazard Ratios from the multivariable Cox regression model further adjusted by pre-diagnosis Body Mass Index and time from diagnosis to second weight assessment

^b^Subdistribution Hazard Ratios from the multivariable Fine and Gray regression model further adjusted by Body Mass Index and time from diagnosis to second weight assessment

### Weight change and mortality

**Fig. 2:**
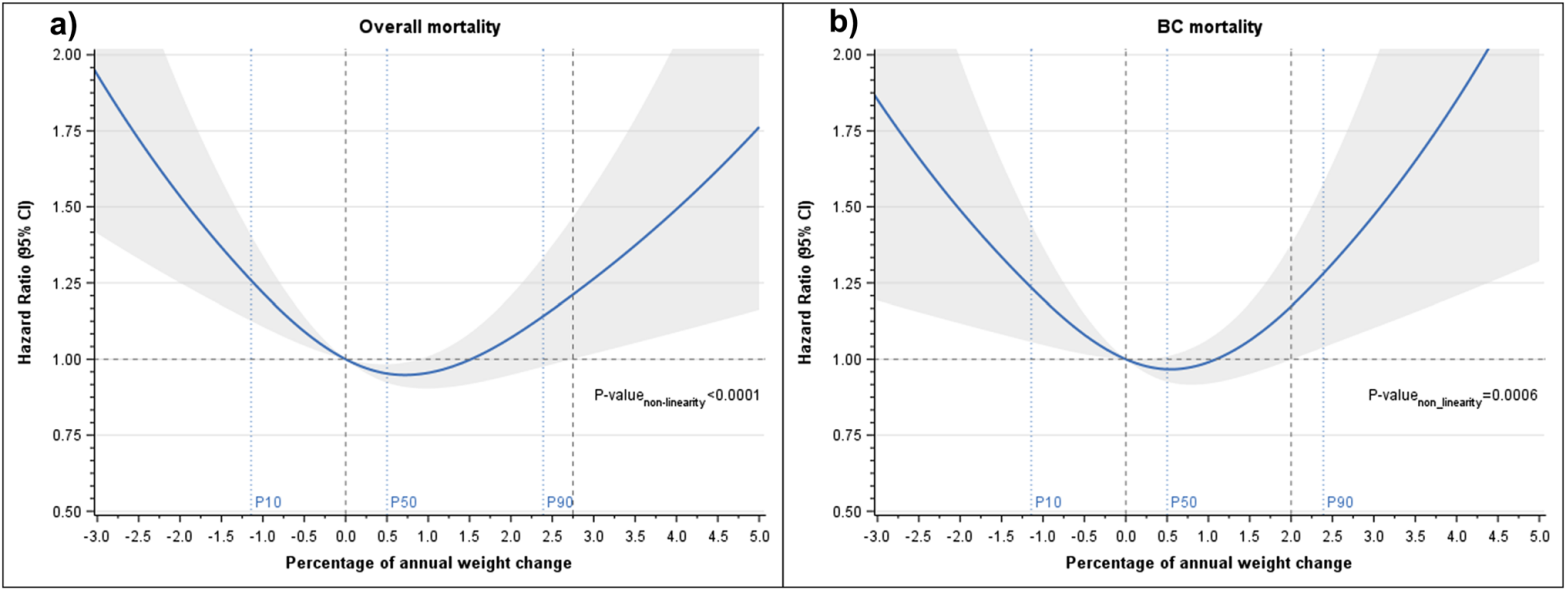
Restricted cubic spline models for post-diagnosis annual percentage of weight change. Footnote: **a** Hazard Ratios from the multivariable restricted cubic spline Cox regression model further adjusted by pre-diagnosis Body Mass Index and time from diagnosis to second weight assessment. **b** Subdistribution Hazard Ratios from the multivariable restricted cubic spline Fine and Gray regression model further adjusted by Body Mass Index and time from diagnosis to second weight assessment. Knot locations were based on Harrell’s recommended percentiles (P10, P50, P90)

The percent annual weight change prediagnosis was not associated with the overall or BC-specific mortality in women with baseline BMI < 25 kg/m^2^ or > 25 kg/m^2^ (Supplementary Table 8). However, it demonstrated a U-shaped relationship with both the overall and BC-specific mortality when the second weight assessment was postdiagnosis (with deviation from linearity, both *p*-values < 0.001), thereby indicating any percentage of weight loss was associated with an increased overall and BC-specific mortality, while the percentage of weight gain revealed an association in the upper range, only for BC-specific mortality (Fig. 2). Due to the observed non-linear association with percentage of annual weight change and mortality, we performed the same survival models to evaluate the association with overall and BC-specific mortality with the percentage variable divided into tertiles, considering the middle tertile as the reference group. The results showed an increase of 45% (13–87%) in the overall mortality for the lowest tertile and an increase of 30% (0–67%) for the highest tertile. We obtained similar estimates for BC-specific mortality, without reaching statistical significance.

### Mendelian randomization

The MR analyses provided estimates similar to those obtained from the observational analyses: the HR for one-unit BMI increase (as predicted by the genetic risk score) was 1.04 (95%CI: 0.90 to 1.20) and 1.04 (0.87 to 1.25) for the overall and BC-specific mortality, respectively (Supplementary Table 9). The corresponding HRs for one-unit increase in the measured BMI were 1.02 (1.01–1.03) and 1.01 (1.00–1.03) for the overall and BC-specific mortality, respectively. However, in the MR analysis, the confidence intervals were wide and none of the effect estimates reached statistical significance.

## Discussion

Survivors of BC with general obesity before diagnosis displayed a 25% and 23% increase in the overall mortality and BC-specific mortality, respectively, compared with women with normal weight. Women with abdominal obesity had also a 22% increased risk of overall mortality. These findings were principally confirmed in postmenopausal women with non-metastatic tumors, in addition to ER + , PR + , and HER2- BCs. The postdiagnosis weight change revealed a U-shaped relationship with the overall and BC-specific mortality, with the highest mortality associated with losing weight, or gaining more than 2% weight annually. The Mendelian randomization analyses were consistent with our observational results.

### Comparison with previous studies

The most recent meta-analysis [2] on the association between prediagnosis general obesity and mortality among patients with BC reported on a 26% and 23% increase in the overall mortality and BC-specific mortality, respectively, for women with obesity than in those without (BMI < 30 kg/m^2^). Apart from different reference categories, these estimates were based on both prospective and retrospective studies. Our estimates were lower than the pooled estimators obtained in a meta-analysis of 82 follow-up studies [6], where obese patients compared with those with normal weight demonstrated a 41% and 35% increase in the overall and BC-specific mortality, respectively. The high estimates of the meta-analysis are possibly due to the influence of studies that include ethnically diverse cohorts.

Few studies have evaluated the association between abdominal obesity and mortality in survivors of BC. We identified the prediagnosis abdominal obesity as an independent risk factor for the overall mortality, in contrast to the findings of several follow-up studies that comprehensively analyzed body fatness and mortality [21].With regards to weight change, patterns of the U-shaped association between postdiagnosis weight change and the overall and BC-specific mortality were consistent with previous studies [22, 23]. Moreover, in the dose–response meta-analyses provided by the Global Cancer Update Programme, although it was not possible to perform an analyses of the shape of the relationship, there was an association with weight loss and higher overall mortality compared with stable weight, and an association with BMI gain with higher overall and BC-specific mortality compared with stable BMI [4]. Our findings were presumably in agreement with the hypothesis that substantial postdiagnosis weight gain is related with poor BC prognosis [24]. Our findings on weight loss also demonstrated similar trend with worse prognosis, despite our inability to distinguish between intentional or unintentional weight loss (owing to cancer treatment or advanced disease).

### Possible biological mechanisms

Several biological mechanisms could explain the effects of excess body fatness on BC mortality and progression [25]. Excess adiposity leads to higher levels of bioavailable fractions of estradiol and testosterone; moreover, in the obese, increased release from adipose tissue of free fatty acids and several endocrine signaling factors, such as tumor-necrosis factor-α, adiponectin, leptin, and resistin, lead to the development of insulin resistance and chronic inflammation. The potential involvement of sex-hormones (principally estrogens) in BC progression may be related with the stronger association of body fatness with the overall and BC-specific mortality in women with ER + tumors.

Survivors of BC with non-metastatic tumors revealed the strongest and significant association between higher body fatness and mortality. Generally, patients with metastatic cancer have poor prognosis, their survival is less likely to be influenced by the long-term effects of factors, such body fatness and body composition, and is substantially determined by the availability and response to treatments.

A huge diversity of circumstances may be associated with pre-diagnostic weight change, intentional weight loss. After diagnosis, weight changes may reflect treatment and disease burden rather than voluntary modification of lifestyle behaviors. It seems that large weight loss, even among overweight/obese women has an adverse rather than protective influence on breast cancer, owing to severe alterations in nutrient intake and absorption that impact energy metabolism. Furthermore, weight loss may be also associated with treatment-related toxicity (reduced appetite, nausea, and diarrhea). Overall, alterations in nutritional and metabolic status may lead to sarcopenia and cachexia, which are strong predictors of worse survival. On the contrary, certain treatment modalities are associated with weight gain, including some chemotherapy regimens and hormonal therapy. The influence of weight change on survival may differ depending on how much time has elapsed since diagnosis. While the association of large weight loss with worse survival seems to be more pronounced earlier in follow-up, in the long-term (several years after diagnosis) women who gain weight experience higher all-cause and breast cancer-specific mortality [23].

There has been increasing interest as to whether environmental exposure to endocrine disrupting chemicals can impact on human health. Many of these chemicals have estrogenic properties, and a large number of studies have confirmed that they are ubiquitously present in human tissues. Some of these chemicals are defined as persistent organic pollutants (POPs) owing to their environmental persistence; they are also highly lipophilic and hence accumulate in adipose tissue. Therefore, there is a complex relationship among obesity, exposure to POPs, and breast cancer. Indeed, POPs can play a role in the biological mechanisms underlying the associations reported in our study, specifically those concerning weight change. POPs are released from adipose tissue into the blood stream when weight is lost, which is often the case in advanced disease or because of alterations in nutritional and metabolic status associated with sarcopenia or cachexia. A systematic review [26] reported that levels of POPs in blood were consistently associated with worse breast cancer prognosis: positive associations with both all-cause and specific mortality were observed, and one study also observed a positive association with breast cancer recurrence.

### Strengths and limitations of the study

One important issue to be considered as a potential limitation of our study was the lack of information regarding the type of treatment, which may be a strong determinant of prognosis. However, the therapeutic management of patients with BC in most settings is strongly determined by the tumor stage at diagnosis and other features, such as grading and receptor status. The adjustment of our models for the aforementioned variables would, at least partially, account for the influence of treatment on survival. Following an adjustment for the tumor stage and subtype by an immune-histochemical analysis, further adjustment for the treatment did not substantially modify the association between nutritional factors and survival [27]. Moreover, our sensitivity analyses adjusted by the period of diagnosis (Supplementary Table 5) confirmed the findings obtained in the primary analyses. Another important limitation was the lack of anthropometric measurements assessed close to the date of diagnosis. Thus, we performed a sensitivity analysis with an additional adjustment by the time from the baseline to diagnosis (Supplementary Table 4), and the results remained unchanged. Conversely, using body fatness measurements recorded during an extended period prior to the diagnosis had the advantage of preventing potential bias induced by the influence of the disease(pre-clinical) or its treatment for measurements recorded near (either before or after) the date of diagnosis [28].

Although our primary analysis was based on a large sample size, we ended up with limited power in the analysis of the postdiagnosis measurements. Only a subset of our participants had a second weight assessment, and only a few of them (20%) had the second measurement done after diagnosis. Moreover, we restricted the analysis to those with second assessments obtained within 6 months and 4 years postdiagnosis; anthropometric measurements collected immediately following the diagnosis may be highly influenced by treatments, whereas those collected considerably later may induce an immortal time bias [29]. Besides, the second weight assessment was self-reported; hence, it was less accurate than the measured weight collected at recruitment. Finally, the lack of information on whether weight change was intentional or unintentional could be a possible limitation.

A limitation of our MR analysis is the potential collider bias [30] by only including BC patients in the analysis. Because the second assumption of the MR analysis could be violated [31], we have checked the genetic instrument remained independent of factors that confound the association of the exposure and the outcome (non-significant p-values were obtained). Also in order to mitigate the potential collider bias we performed the MR including stage and grade of the disease in the model analysis as a rudimentary method to asses this bias (Supplementary material).

The major strengths of the study were its prospective design, large sample size, long follow-up, and the availability of detailed and validated information on exposure, outcomes, and most relevant confounders. To our knowledge, this is the first study to include, within a particular cohort, an assessment of the independent relation between mortality and both general and abdominal obesity, the evaluation of pre- and postdiagnosis measures of body fatness and weight change in relation with prognosis, the evaluation of these relationships according to the menopausal status, tumor stage, and receptors, and an approach to assess the causal relationship between the BMI and mortality by means of an MR analysis. The independent assessment of two indicators of different forms of body fatness was particularly relevant. General and abdominal obesity is often evaluated based on the BMI and WC, respectively. However, these measures are highly correlated, thus precluding the assessment of their independent association with mutual adjustment. Instead, we used the ABSI, which is specifically designed as uncorrelated from BMI, thereby allowing the inclusion of both measures in the identical model without a risk of bias [11]. Considering the complex nature of body fatness, researchers should use an indicator of abdominal adiposity to complement rather replace the BMI. This is because none of them in isolation adequately reflect the associations of both the body size and shape.

## Conclusions

In conclusion, our findings supported the association between body fatness and poorer survival among BC cases in this observational prospective cohort. Furthermore, they supported earlier findings that substantial weight changes following diagnosis (both loss and gain) may be associated with poorer survival. Taken together, our results add to existing evidence encouraging the development of lifestyle recommendations for breast cancer patients aimed to maintaining a healthy weight, along with further research to determine the most effective strategies to increase adherence to these recommendations. Well-designed intervention and observational studies, as well as mechanistic studies, are needed to elucidate the impact of body composition and distribution (beyond body mass index and weight) on the prognosis of breast cancers survivors. The outcomes of such studies should include, in addition to the mortality, the risk of recurrence and quality of life of survivors. Specifically, intervention trials are needed to elucidate whether sustainable, intentional weight loss can reverse the adverse pathological effects and improve survival outcomes in breast cancer patients with overweight and obesity.

## Supplementary Information

Below is the link to the electronic supplementary material.

Supplementary file1 (DOCX 22 KB)

Supplementary file2 (XLSX 220 KB)
