# Supplementary material for: The association between body fatness and mortality among breast cancer survivors: results from a prospective cohort study
Source: Eur J Epidemiol. 2023 Mar 29;38(5):545–57. doi: 10.1007/s10654-023-00979-5 (PMC10163997; doi:10.1007/s10654-023-00979-5)
Supplement: Supplementary file 1 — Supplementary file1 (DOCX 22 KB) [file 10654_2023_979_MOESM1_ESM.docx]

**Supplementary material**

*Survival models*

Hazard ratios (HR) and 95% confidence intervals (CI) to assess the associations between the exposures (anthropometric measures used as markers of body fatness) and mortality outcomes were derived from multivariate Cox proportional hazards (PH) regression models for overall mortality, and from Fine-Gray competing risk models for breast cancer-specific mortality. Depending on whether the anthropometric data under analysis were measured at baseline or after diagnosis, the considered time-scale was time since diagnosis of breast cancer (BC) or time since the second anthropometric assessment; in both cases, exit time was the date of death, emigration or end of follow-up, whichever came first. The survival models were stratified by country and menopausal status at diagnosis. Menopausal status at diagnosis was derived from the information collected at baseline updated with the information of the second assessment gathered during the follow-up. Women with age at diagnosis >55 years were considered postmenopausal, independently of the information collected at recruitment. The survival models were adjusted for the following potential confounders: age at diagnosis (5-years categories); attained level of education (none, primary school, secondary school, technical/professional school, longer education, unknown); physical activity (inactive, moderately inactive, moderately active, active, unknown); alcohol consumption (grams/day) reported at recruitment (nondrinker, >0-3 , >3-12, >12-24,>24, unknown); smoking habit and intensity as cigarettes per day (cig/d) at recruitment (never, current 1-15cig/d, current 16-25 cig/d, current >26 cig/d, former quitted 10 years before recruitment, former quitted 11-20 years before recruitment, former quitted more than 20 years before recruitment, current smoker of cigars, pipes and occasional current smokers, current smokers with missing information on the intensity, unknown); ever use of hormone for menopause at diagnosis (yes, no, unknown); tumor stage at diagnosis (0/I, II, III, IV, non-metastatic but unknown specific stage, and unknown); tumor grade (well differentiated, moderately differentiated, poorly differentiated or undifferentiated, not determined); and tumor receptor status: ER (+,-,unknown), PR(+,-,unknown), HER2 (+,-,unknown). In order to avoid losing power in the analyses, a missing indicator category was created for each of the categorical covariate with missing values. The proportional hazards assumption was evaluated by checking the graphs of the scaled Schoenfeld residuals.

When the main exposure (marker of body fatness) was treated as a continuous variable, restricted cubic splines models were used to check the validity of the assumed log-linear dose-response association with the mortality outcomes; the non-linearity was tested by means of the Likelihood Ratio test. We used 3 knots to construct the spline term of the model, knot locations were based on Harrell’s percentiles.

*Mendelian randomization analysis*

GWAS data available for 8,494 participants of EPIC (among which 3,830 were breast cancer cases) were used to perform a Mendelian randomization (MR) analysis. Part of the GWAS genotyping was conducted using Ilumina Human 660k-Quad SNP array (Illumina, Inc.) and part with Infinum OncoArray-500k. Exclusion criteria were: genotyping sample call rate <90%, SNP call rate <90%, or cryptic relatedness (>80% concordance). Imputation of genetic variants was performed by using the Michigan Imputation Server, according to established guidelines (https://imputationserver.sph.umich.edu) using the haplotype reference panel 1000 Genomes Phase 3 (Version 5).

The 94 BMI-associated SNPs used to construct the weighted genetic risk score (wGRS) are displayed on eTable 9 in the Supplement. Based on the genetic effect estimates on BMI reported by Locke et al. (2015), each SNP was coded so that the reference allele was associated with an increase in BMI. The SNPs were weighted by its relative effect size on BMI and then summed across all variants. The result was then divided by the total effect size of all SNPs and multiplied by the number of the SNPs (n=94). Therefore, the wGRS represents the number of average BMI increasing alleles possessed by each breast cancer survivor. In our study, each unit increase in the wGRS was associated with 0.08 (95% CI 0.06-0.10) kg/m^2^ increase in BMI, explaining 1.55% of the variance.

There is not a gold standard MR estimator in the context of cohort studies and survival analyses (Cho et al. 2021); therefore, we used a methodology similar to Wade et al. (2018). Firstly, the BMI was linearly regressed on the wGRS among the subset of non-BC participants with GWAS data (n=4,664) adjusting by country and age at recruitment. Second, the log(HR) of mortality among breast cancer cases per each unit increase of the wGRS was derived from a Cox PH model adjusted by country, age at diagnosis, and by stage and grade of the tumor to avoid possible collider bias (Mitchell et al. 2018, Paternoster et al. 2017). Finally the instrumental variable (IV) estimator was calculated exponentiating the ratio between the log(HR) of mortality with each unit increase of the wGRS and the slope coefficient of the linear regression of wGRS with BMI. The 95% CI of the IV was obtained with the delta-method using Taylor series expansion (Thomas et al. 2007). In order to judge the weakness of the IV we extracted the mean coefficient of determination $R^{2}$ from the regression of BMI on the wGRS.

Sensitivity analyses were carried out to: i) check the non-violation of the MR assumptions, ii) check PH assumption, and iii) check the linearity of the wGRS with the mortality outcomes. To check the non-violation of the MR assumptions, we first studied the validity of the wGRS as an IV using the MR-Egger (Bowden et al. 2015), weighted median and Maximum-likelihood based estimators compared with the inverse-variance weighted method for two sample MR (Table S7), then we tested the association between wGRS and the covariates (no associations were found, results not shown), and finally we explored the sensitivity of the wGRS by the leave-one-out method applied on the SNPs. The PH assumptions were examined with the Schoenfeld residuals, and the linearity of the association was studied with restricted cubic splines.

References used in this section:

Locke AE, Kahali B, Berndt SI, Justice AE, Pers TH, Day FR, et al. Genetic studies of body mass index yield new insights for obesity biology. Nature. 2015 Feb 12;518(7538):197–206.

Cho Y, Rau A, Reiner A, Auer PL. Mendelian randomization analysis with survival outcomes. Genet Epidemiol. 2021 Feb;45(1):16–23.

Wade KH, Carslake D, Sattar N, Davey Smith G, Timpson NJ. BMI and Mortality in UK Biobank: Revised Estimates Using Mendelian Randomization. Obes Silver Spring Md. 2018 Nov;26(11):1796–806.

Mitchell RE, Paternoster L, Davey Smith G. Mendelian Randomization in Case Only Studies: A Promising Approach to be Applied With Caution. *Am J Cardiol* 2018;**122**:2169–71. doi:10.1016/j.amjcard.2018.09.035.

Paternoster L, Tilling K, Smith GD. Genetic epidemiology and Mendelian randomization for informing disease therapeutics: Conceptual and methodological challenges. *PLOS Genet* 2017;**13**:e1006944. doi:10.1371/journal.pgen.100694.

Thomas DC, Lawlor DA, Thompson JR. Re: Estimation of bias in nongenetic observational studies using ‘Mendelian triangulation’ by Bautista et al. Ann Epidemiol. 2007 Jul;17(7):511–3.

Bowden J, Davey Smith G, Burgess S. Mendelian randomization with invalid instruments: effect estimation and bias detection through Egger regression. Int J Epidemiol. 2015 Apr;44(2):512–25.
